# Diagnostics for detection and surveillance of priority epidemic-prone diseases in Africa: an assessment of testing capacity and laboratory strengthening needs

**DOI:** 10.3389/fpubh.2024.1438334

**Published:** 2024-09-18

**Authors:** Aytenew Ashenafi, Olajumoke Sule, Trevor Peter, Silver Mashate, Osborn Otieno, Abebaw Kebede, John Oio, Kekeletso Kao, Jane Carter, Toni Whistler, Nqobile Ndlovu, Yenew Kebede

**Affiliations:** ^1^Centre of Laboratory Systems Division, Africa Centres for Disease Control and Prevention, Addis Ababa, Ethiopia; ^2^International Health Regulations Strengthening Project, Health Protection Operations, United Kingdom Health Security Agency, London, United Kingdom; ^3^Clinton Health Access Initiative, Boston, MA, United States; ^4^African Society for Laboratory Medicine, Addis Ababa, Ethiopia; ^5^Technical Advice and Partnerships Department, The Global Fund to Fight AIDS, Tuberculosis and Malaria, Geneva, Switzerland; ^6^Diagnostic System Strengthening Unit, FIND, Geneva, Switzerland; ^7^Clinical and Diagnostics Programme, Amref Health Africa, Nairobi, Kenya

**Keywords:** public health emergencies, laboratory testing capacity, epidemic-prone diseases, outbreak response, cost-effective diagnostic technologies

## Abstract

In 2023, Africa experienced 180 public health emergencies, of which 90% were infectious diseases and 75% were related to zoonotic diseases. Testing capacity for epidemic-prone diseases is essential to enable rapid and accurate identification of causative agents, and for action to prevent disease spread. Moreover, testing is pivotal in monitoring disease transmission, evaluating public health interventions and informing targeted resource allocation during outbreaks. An online, self-assessment survey was conducted in African Union Member States to identify major challenges in testing for epidemic-prone diseases. The survey assessed current capacity for diagnosing priority epidemic-prone diseases at different laboratory levels. It explored challenges in establishing and maintaining testing capacity to improve outbreak response and mitigate public health impact. Survey data analysed diagnostic capacity for priority infectious diseases, diagnostic technologies in use, existing surveillance programmes and challenges limiting diagnostic capacity, by country. The survey result from 15 Member States who responded to the survey, showed high variability in testing capacity and technologies across countries and diverse factors limiting testing capacity for certain priority diseases like dengue and Crimean-Congo haemorrhagic fever. At the same time diagnostic capacity is better for coronavirus disease 2019 (COVID-19), polio, and measles due to previous investments. Unfortunately, many countries are not utilizing multiplex testing, despite its potential to improve diagnostic access. The challenges of limited laboratory capacity for testing future outbreaks are indeed significant. Recent disease outbreaks in Africa have underscored the urgent need to strengthen diagnostic capacity and introduce cost-effective technologies. Small sample sizes and differing disease prioritisation within each country limited the analysis. These findings suggest the benefits of evaluating laboratory testing capacity for epidemic-prone diseases and highlight the importance of effectively addressing challenges to detect diseases and prevent future pandemics.

## Introduction

In 2023, Africa has documented more than 168 public health events affecting 45 African Union Member States mostly infectious disease outbreaks including mpox, diphtheria, dengue, Lassa fever, measles, poliomyelitis, Rift Valley fever and cholera. These outbreaks continue to expand rapidly and affect many countries on the continent. Since the beginning of 2024, a total of 26,122 cholera cases (3,396 confirmed; 22,726 suspected) with 663 deaths were reported from 12 African countries; 4,451 cases of measles (178 confirmed; 4,273 suspected) and 51 deaths were reported from seven African countries; and for diphtheria 1,573 cases were reported (904 confirmed, 669 suspected) with four deaths from two African countries (1–4).

The International Health Regulations (IHR, 2005) identify disease mapping, health risk assessment and resource prioritisation as among the core capabilities of public health emergency preparedness and response systems (5). Recurrent outbreaks of emerging and re-emerging pathogens have revealed major gaps in these systems in countries on the African continent. Key mechanisms to prevent recurrent disease outbreaks include early detection of infectious diseases in the community, rapid pathogen identification, and integrated disease surveillance. Surveillance and response capacity is limited in many African countries, especially in the context of COVID-19, Ebola virus disease (EVD), and in detection and monitoring of antimicrobial resistance (6). As the recent EVD, COVID-19 and mpox outbreaks highlight, a major paradigm shift is required to establish effective infrastructure and mechanisms for outbreak detection, and common frameworks for preparedness and national public health responses to manage future epidemics (7).

The Africa Centres for Disease Control and Prevention (Africa CDC) has drawn up a preliminary list of priority epidemic-prone diseases for Africa using a risk ranking and analysis tool to identify the target infectious diseases for epidemic preparedness and response actions (8), and to inform research and technology development and disease control innovations such as development and provision of vaccines, diagnostics, and therapeutics. For example, EVD, cholera and COVID-19 scored the highest for disease severity, risk and epidemic potential, as well as the need for preparedness through vaccine availability, and medical and non-medical countermeasures. The disease ranking process changes every time as more evidence comes in.

The ability to respond effectively to disease outbreaks depends on several factors, including laboratory capacity, a trained health workforce, and robust surveillance systems (9), which are public health systems that rapidly diagnose and contain the spread of infectious diseases. While significant diagnostic capacity has been established over the past two decades for major endemic diseases such as HIV/AIDS, tuberculosis (TB), malaria and more recently COVID-19, testing capacity for other epidemic-prone infections is more limited. Therefore, a comprehensive approach is needed to address the gaps in pandemic preparedness and health systems’ resilience (10, 11).

The Africa CDC undertook a survey of African Union Member States on disease priorities, laboratory capacities and public surveillance systems to help identify major challenges faced in building testing capacity for outbreak detection and response for epidemic-prone diseases in African countries. The findings of this survey are presented in this paper.

## Methods

### Survey description

All 55 African Union Member States were invited to participate in a self-assessment survey of laboratory testing capacity and surveillance of 22 epidemic-prone diseases between February and April 2023. In 2022, the Africa CDC, in partnership with European Center for Disease Prevention and Control (ECDC), conducted a risk ranking and prioritization exercise identifying diseases in Africa that are prone to epidemics and require immediate and effective responses. This initiative employed criteria to categorize and rank these diseases into four main groups, encompassing risk trajectory, epidemic potential, disease severity, and the preparedness and countermeasures levels in place (8).

### Survey instrument

The development of the survey tool involved a rigorous validation process to ensure its reliability and accuracy in measuring the intended objectives. Initially, a draft of the survey questionnaire was evaluated by experts who provided insights on the relevance, clarity, and thoroughness of the questions. Based on their feedback, the questions were subsequently revised to guarantee that the survey adequately addressed all dimensions of the concept under investigation. After these revisions, the survey was administered to a selected group of respondents for a final testing phase. In consultation with members of the Africa Laboratory Technical Working Group the survey was refined and modified based on the results obtained (Supplementary material S1). It included questions on disease testing capacity, technology in use, healthcare system level and presence of surveillance programmes. Additionally, questions addressed multiplex testing capacity and challenges faced.

Participants ranked their capacity to diagnose the priority epidemic-prone diseases (Supplementary material S2) on a five-point Likert scale (from 1 to 5), with 5 being the highest. Quantitative questions included information on disease testing capacity across countries’ healthcare systems, standard methods used to diagnose diseases (PCR, multiplex PCR, lateral flow assays, ELISA, etc.), biosecurity, sample transport, data systems, staffing and existing surveillance programmes. Qualitative questions were also included to allow respondents to add relevant comments, and to identify countries’ most significant challenges and the support needed to ensure adequate diagnostic capacity for future outbreaks and epidemics. The survey tool was developed in a Microsoft Excel format using dropdown lists to make it user-friendly. The tool was provided in English and French, pre-tested, amended and distributed online to respondents.

The first section of the survey comprised 8- questions for disease testing capacity to correlate the epidemic-prone disease to the capacity of diagnosis and technology in use and surveillance programmes for these diseases. The respondent completed the questions. Some with a dropdown menu to for answer selection. The tool allows countries to add a comment describing their testing method and asking about the standard methods used to diagnose diseases (PCR, Multiplex PCR, Lateral Flow Assay, ELISA, and others). Based on the responses, the ability to diagnose the twenty-two-priority epidemic-prone diseases was rated on a five-point scale from 1 to 5, with 5 representing the highest score.

The second survey has 3-questions for multiplex testing capacity and panel of multiplex testing, and levels of laboratory testing capacity across Member States’ healthcare systems.

The third survey had 8-miscellaneous and 3-qualitative questions on sample transport, biosecurity, data systems and staffing. Some qualitative questions were included to get a general sense of the support countries need/want and explore countries’ biggest challenges to ensuring diagnostic capacity to be ready for a future epidemic.

### Dissemination of the survey tool to countries and data collection

All African countries were invited to participate in the survey, targeting national public health laboratory directors, managers and heads of reference laboratories. We collected the informed consent form from online survey links obtained from a network of laboratories that got a request to participate in a survey. Data collection was from February to April 2023. We implemented a number of techniques to boost study participation. First, an email outlining the significance and applicability of the study was sent to prospective respondents, informing them of the impending survey. After that, participants received follow-up emails regularly to remind them to complete the survey. Furthermore, we reassured participants about the confidentiality of their responses, which helped to build trust and increased their willingness to participate in the survey.

### Data entry and analysis

The questionnaires aligned with the survey objectives and underwent testing for clarity and functionality. After distribution and collection, the data was cleared to eliminate duplicates, rectify errors, and address incomplete responses. Subsequently, qualitative responses were categorized and assigned numerical values to facilitate analysis. Data were entered into SPSS 2023 and Microsoft Excel for analysis, visualisation, and results interpreted to draw meaningful conclusion and identify findings in line with the research objectives. Finally, the results were compiled into comprehensive reports, complete with visualisations and actionable recommendations to effectively communicate the insights gained to the technical working group.

### Ethics considerations

Ethics approval was not sought for the following reasons.

The survey pertained to member state diagnostic capacity and did not include data on individual patients or vulnerable people. The survey instrument was developed in consultation with members of Africa CDC Laboratory Technical Working Group. Participation by member states was voluntary following informed consent. The survey utilized an online methodology, ensuring that the results were anonymized and could not be tracked back to individual member states. Responses were stored securely on an Africa CDC sever. The Africa operates solely within the African continent and provides a platform that offers secure data storage solutions tailored to meet regional security requirements. This platform employs robust encryption, access controls, and monitoring systems to safeguard survey responses and keep confidentiality. Furthermore, permission settings were implemented to manage access to survey data, thereby protecting confidentiality.

## Results

### Survey respondents

**Figure 1:**
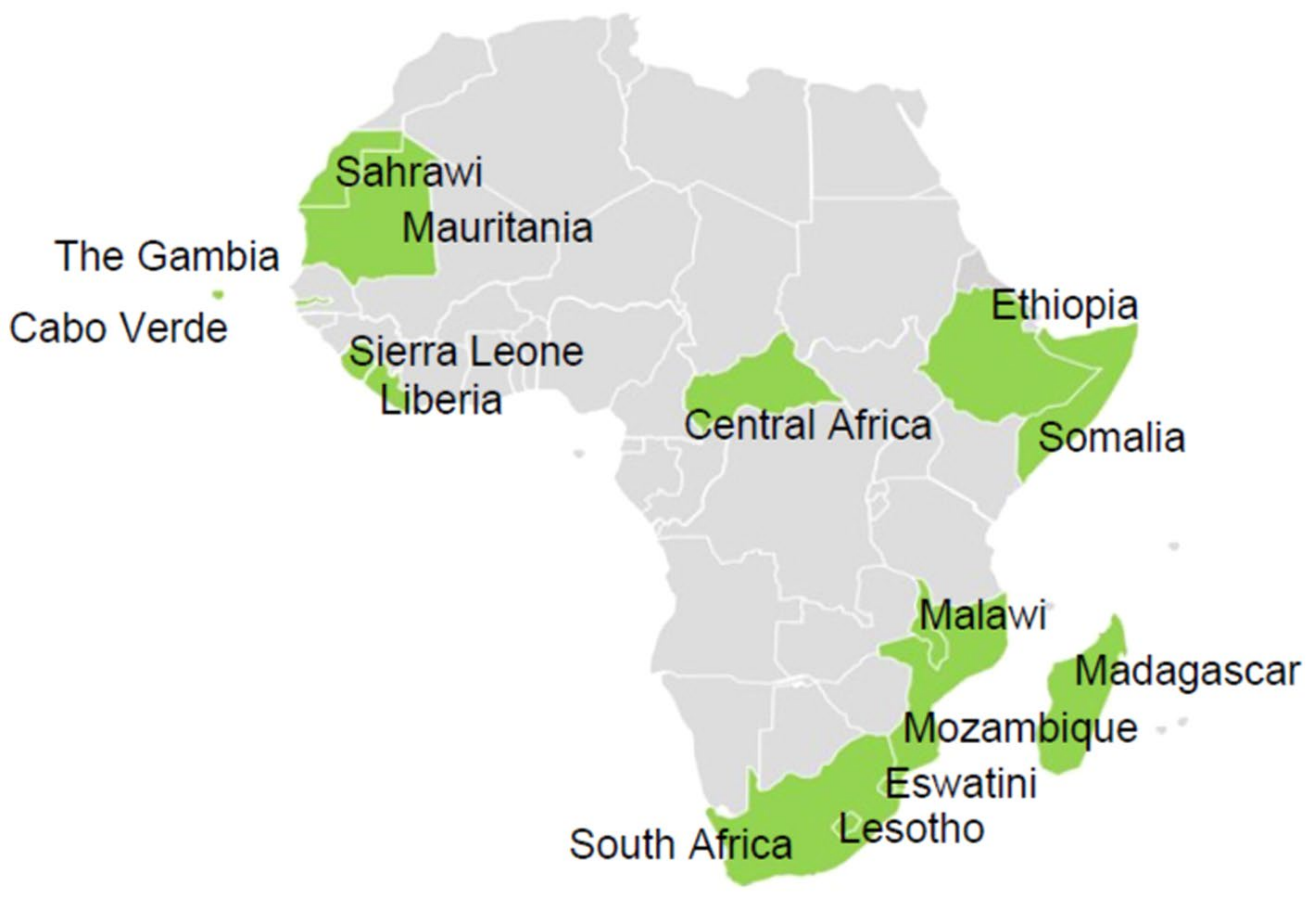
Map showing countries that participated in the survey (*n* = 15).

Participants from 15 of 55 (27%) Member States responded to the survey (Figure 1). Respondents were distributed as follows: five (50%) from the southern region, four (27%) from the western region, three (21%) from the eastern region, two (29%) from the northern region, and one (11%) from the central region. The survey was completed by laboratory directors (7) and senior technical staff members (8) working at either national public health institutes (12) or ministries of health (3).

### Disease testing capacity by laboratory level

**Figure 2:**
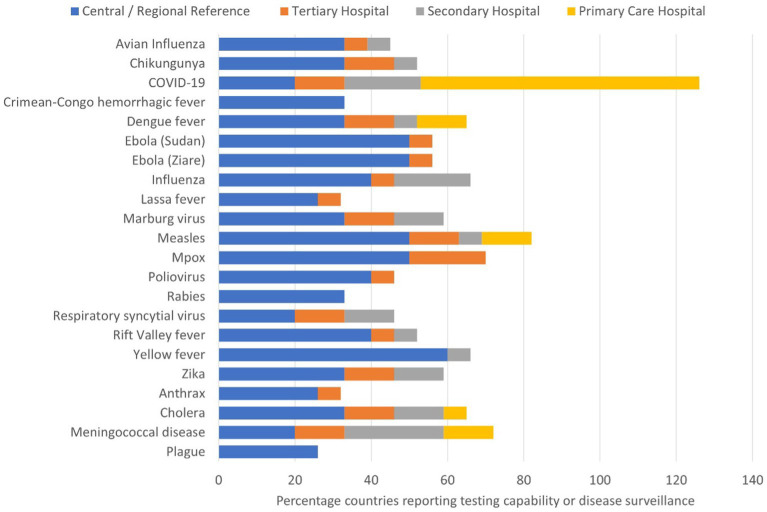
Stacked bar graph showing the percentage of countries (*n* = 15) that can test for the specified priority epidemic-prone disease at each tier of the laboratory system. Tires were divided into central or regional reference laboratory, and tertiary, secondary and primary care hospital laboratories.

Capacity for diagnosing infectious diseases varied based on the disease, country and laboratory level (Figure 2). Testing for viral haemorrhagic fevers was conducted at central public health laboratories and tertiary care hospital laboratories, while testing for COVID-19, meningococcal meningitis, cholera, measles and dengue was conducted at laboratories ranging from reference to district level.

The most common testing technology reported for diagnosing causative agents of epidemic-prone diseases was real-time polymerase chain reaction (qPCR) followed by enzyme-linked immunosorbent assay (ELISA). There were differences in testing capacity and technology across the participating countries. All 15 countries reported use of PCR testing for COVID-19. Measles and mpox were the next most common diseases diagnosed by PCR. Among Member States using PCR for testing, open-source instruments were available in 60% of national public health laboratories, 47% of regional and tertiary laboratories, and 20% of secondary care laboratories. ELISA was routinely performed to diagnose seven diseases (measles, yellow fever, CCHF, Rift Valley fever, dengue, Zika and chikungunya) across 11 countries, with measles being the most common. Among bacterial infections, few countries reported using molecular techniques for diagnosis, with between one and three countries each reporting molecular techniques for diagnosing cholera, plague, anthrax, and meningococcal disease. Two countries reported conducting culture for diagnosing cholera, plague, and meningococcal disease. Some countries did not report diagnostic capacity for testing certain infections. Eleven of the 15 countries did not test for anthrax, and 10 countries did not perform testing for plague and rabies due to lack of capacity.

### Surveillance programmes for priority infectious diseases

**Figure 3:**
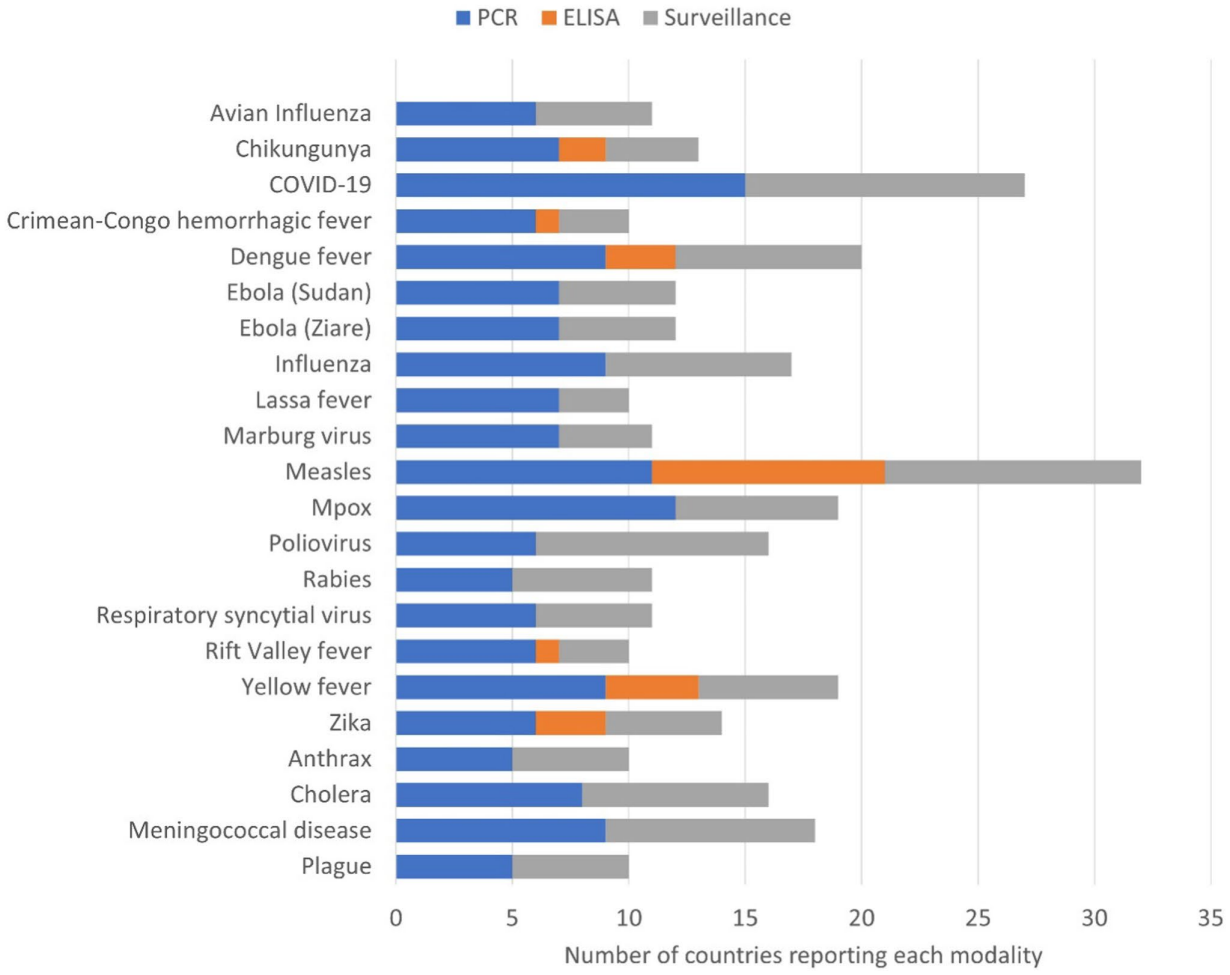
Number of countries (*n* = 15) reporting capacity for laboratory diagnosis and current active surveillance systems. Diagnostic capacity was divided into molecular tests using polymerase chain reaction (PCR) and/or Enzyme linked immunosorbent assay (ELISA) of the priority epidemic-prone diseases and current active surveillance systems.

The three most common surveillance programmes that countries were actively running during the survey were COVID-19, measles, and polio (Figure 3). Twelve countries reported having surveillance programmes for between three and 12 priority diseases. Several countries reported both the availability of PCR testing and the presence of surveillance programmes for some priority diseases, for example, plague, anthrax, cholera and meningitis. For polio, 10 countries reported having surveillance programmes, but only six countries indicated they have PCR diagnostic capacity.

### Epidemic-prone disease priorities for diagnosis and surveillance

**Figure 4:**
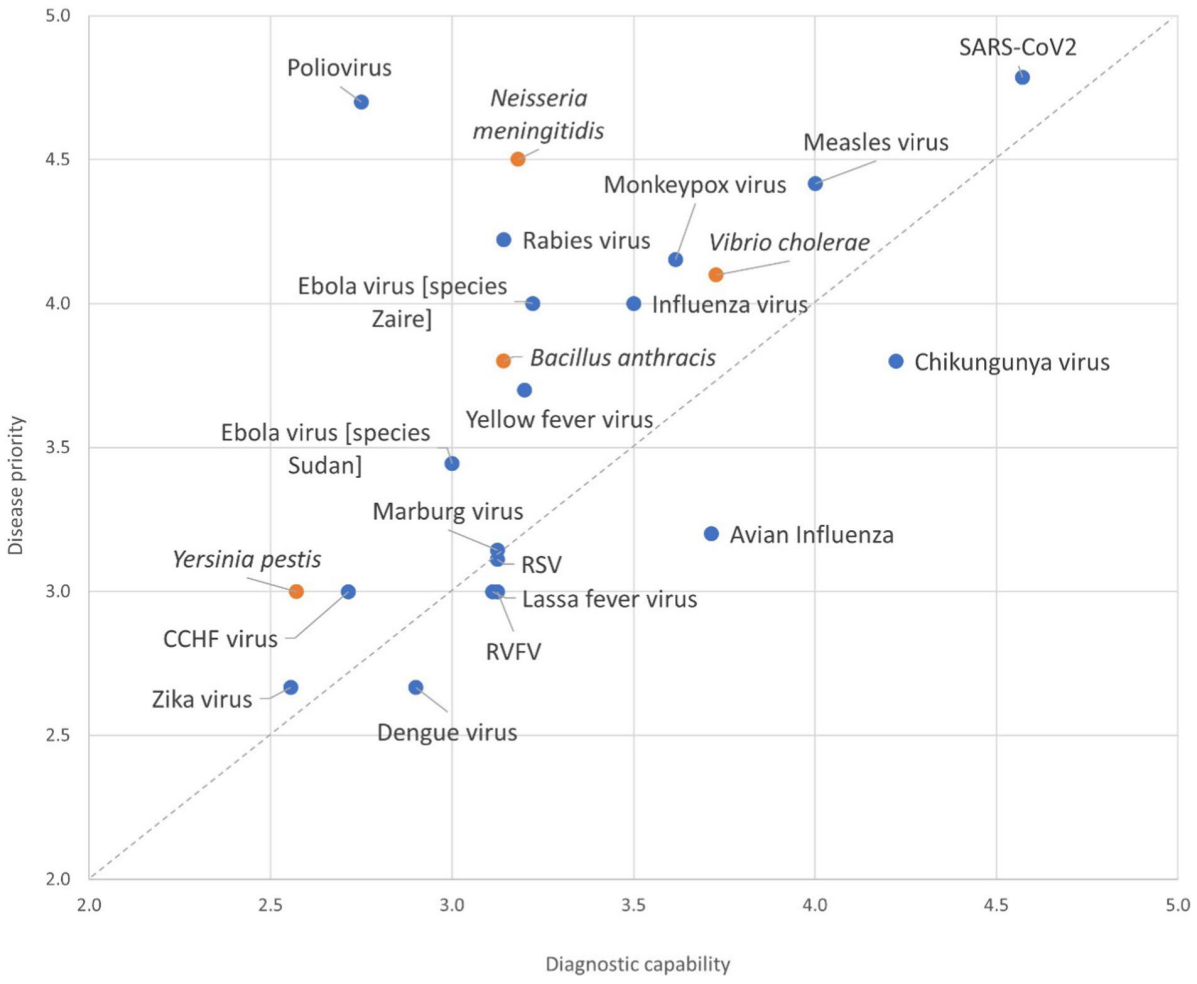
Self-reported testing capacity against the country priority for each epidemic-prone disease across the 15 Member States participating in the survey. Blue marker represents viral diseases, and the orange marker designates bacterial diseases. Inputs were provided on a Likert scale (1–5, with 5 being the highest priority), the average scores across reporting countries for the pathogen are plotted. CCHF Crimiean Congo Hemorrhagic Fever; RSV Respiratory Syncytial Virus; RVFV Rift Valley Fever Virus.

There were large differences in countries’ priorities for disease diagnosis and surveillance. Thirteen countries reported COVID-19 as the highest priority for their country, whereas two countries reported dengue fever as their highest priority. Others reported avian influenza, plague, anthrax, respiratory syncytial virus (RSV) disease, chikungunya, and EVD as their priority. There were disparities between priority and the capacity to diagnose diseases. For example, while COVID-19 was reported to be a high-priority disease with high diagnostic capacity among countries, four countries reported polio as a high-priority disease but with low diagnostic capacity using the Likert scale 1–5 for both capacity to diagnosis and priority (Figure 4).

### Need for laboratory multiplexing testing capacity

Most respondents indicated the need for multiplex diagnostic testing capacity for infectious respiratory illnesses and arbovirus infections followed by meningitis (Table 1). For some diseases, the perceived need was less commonly reported.

**Table 1:** Number of countries (*n* = 14) reporting the need for multiplex testing by disease and/or pathogens.

Diseases (pathogens) for which multiplex testing was reported as most needed	Number of countries *n* = 14 (%)
Infectious respiratory Illness (influenza virus, RSV, SARS-CoV-2)	11 (79%)
Arbovirus (dengue, chikungunya, Zika, yellow fever, Rift Valley fever, West Nile viruses, encephalitis)	9 (64%)
Meningitis (including *Neisseria meningitidis*, *Streptococcus pneumoniae*)	7 (50%)
Diarrhoeal illness including cholera	3 (21%)
Viral haemorrhagic fevers (including CCHF virus, Marburg virus, Lassa virus)	3 (21%)

Member States were asked which diseases they felt in need of multiplex capacity and responses were grouped by disease or pathogen.

### Reported challenges limiting diagnostic capacity for future epidemics

**Figure 5:**
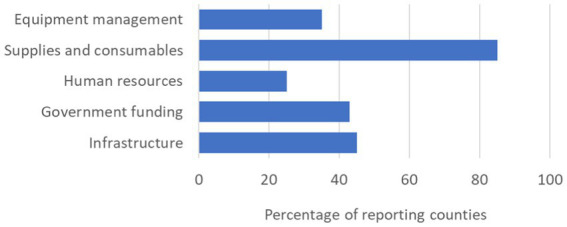
Reported gaps in laboratory diagnostics capacity limiting support for future epidemics.

Eighty-five percent of countries that responded to the survey cited inconsistent laboratory supplies such as PCR reagents, extraction kits and consumables as the primary challenge to developing laboratory capacity to diagnose future epidemics. This was followed by inadequate infrastructure (45%), limited government funding (43%), inadequate equipment management (35%) and inadequate human resources (25%) (Figure 5).

## Discussion

The survey aimed to understand countries’ laboratory diagnostic needs for epidemic preparedness and response. The results offered insights into existing laboratory capacity for priority epidemic-prone diseases across 15 countries. They provided an overview of the capacity within functional tiered laboratory networks across various African Union Member States. These capacities are crucial for implementing the IHR and addressing urgent public health threats such as COVID-19, EVD, measles, CCHF, and others.

In recent years, the Africa region has experienced a surge in outbreaks and epidemics, threatening the health of populations and socio-economic stability, with potential global ramifications (12). Reported disease outbreaks include yellow fever, Rift Valley fever, mpox, measles, CCHF, dengue fever, chikungunya, EVD, Marburg virus disease, cholera, diphtheria, and COVID-19. However, Africa’s inadequate disease diagnosis and surveillance systems hinder the prediction, prevention and effective management of emerging infectious diseases (13). Delayed detection of outbreaks is attributed to a lack of real-time surveillance, suboptimal laboratory networks, limited diagnostic capabilities, inadequate resources, and insufficient technical and managerial capacities.

This survey showed that the capacity for diagnosing infectious diseases varied by disease, country, and laboratory level within tiered networks. The most common technology for diagnosing epidemic-prone diseases was PCR, followed by ELISA. All 15 participating countries reported the use of PCR testing for COVID-19, highlighting the improvements in diagnostics from the COVID-19 pandemic. In contrast, priority diseases such as polio and plague had limited PCR testing capacity. These results may reflect differing country priorities, levels of laboratory testing maturity, and/or the availability of national and external funds to diagnose priority pathogens. The findings suggest that policy and decision-makers have shown a consistent commitment to improving laboratory capacity and system development, as demonstrated by the high capacity to diagnose COVID-19; however, our assessment has also uncovered significant deficiencies in disease diagnostic capacity, particularly at different levels of the tiered laboratory networks that handle epidemic-prone diseases.

Lower-level laboratories exhibited weaknesses in testing for diseases such as Rift Valley fever, CCHF, plague, dengue fever and Zika, which were predominantly conducted at central and regional laboratories, compared to their capabilities for diagnosing COVID-19, polio, measles, mpox, cholera and meningitis. This disparity reflects historical prioritisation and investment in testing for specific diseases, technological advancements, biosafety concerns, and the diversity and availability of funding resources. The COVID-19 crisis underscores the world’s historical unpreparedness to detect and respond to emerging infectious diseases (14). Recent outbreaks of diseases such as Lassa fever, EVD, Marburg virus disease, diphtheria, anthrax, and cholera across the continent highlight the urgent need to strengthen diagnostic capacity for emergency response and outbreak management. Lassa fever’s expanding geographical spread in West Africa underscores the evolving epidemiology of infectious diseases and the necessity for all countries to possess resilient and adaptable diagnostic capabilities (15).

Inadequate diagnostic testing remains a significant issue in African countries (16, 17), particularly at lower-level laboratories. Many commercially available diagnostic tools do not meet the needs of these laboratories or cannot be used due to weak infrastructure. Increased investment in testing capacity at lower-level laboratories is crucial, along with developing and implementing appropriate technologies for diagnosing epidemic-prone diseases. Some tests are available in multiplex format, enabling the detection of multiple pathogens in a single sample for both surveillance and clinical management purposes. Establishing diagnostic capacity for priority diseases of epidemic potential through integrated testing within existing laboratory capacity and with multiplex testing will improve epidemic preparedness, aid early detection and response, and help prevent outbreaks from becoming pandemics (18, 19).

Limited access to diagnostic testing has been a bottleneck in the early detection of priority diseases in Africa (20–22). Diagnostic capacity has often focused on diseases such as HIV, TB, malaria, and more recently, COVID-19 (23, 24). However, laboratories and health systems established for these infections can also be used to detect other priority diseases of epidemic potential, thereby expanding the range of pathogen detection capacity in African Union Member States using existing methodologies.

This survey highlights that the effectiveness of response measures is impeded by various factors, including limited laboratory capacities, difficulties in accessing diagnostic services, shortages of human resources, and fragmented surveillance systems. These challenges exacerbate the complexities of response efforts in numerous countries within the Africa region. The integration of testing and effective use of existing diagnostic platforms and laboratory infrastructure were demonstrated with SARS-CoV-2 testing during the COVID-19 pandemic.

The limitations of this survey included reliance on self-reporting and the limited number of countries that responded. Despite these limitations, the survey results provided valuable insights into diagnostic capacity, disease priorities, surveillance systems and current challenges to the diagnosis of epidemic-prone diseases and has highlighted gaps that urgently need to be addressed to enhance epidemic preparedness, facilitate early disease detection and response, and mitigate the risk of outbreaks escalating into pandemics.

## Conclusion

The increased technical capacity for diagnosing infectious diseases since the COVID-19 epidemic is evident. With ongoing outbreaks of diseases such as yellow fever, Rift Valley fever, mpox, measles, CCHF, dengue fever, chikungunya, EVD, Marburg virus disease, cholera and diphtheria across the continent, there is an urgent need to address the gaps in diagnostic capacity and disease surveillance. Recognising the varying country priorities, expanding point-of-care and multiplex testing for syndromic disease surveillance, and monitoring changes in epidemiology are crucial steps to enhance readiness for detecting epidemic-prone diseases. Countries need to prioritise rapid diagnostic tests and portable technologies while strengthening national and regional laboratory networks. This requires updating infrastructure, improving the laboratory supply chain, and investing in staff training and development.

## Data Availability

The original contributions presented in the study are included in the article/Supplementary material, further inquiries can be directed to the corresponding author.

## References

[1] World Economic Forum. Building resilient healthcare systems in Africa with a focus on outbreaks and epidemics (2023). Available at: https://www.weforum.org/agenda/2023/07/building-resilient-healthcare-systems-in-africa-with-a-focus-on-outbreaks-and-epidemics/ (Accessed February 5-12, 2024).

[2] Moyo E Mhango M Moyo P Dzinamarira T Chitungo I Murewanhema G. Emerging infectious disease outbreaks in sub-Saharan Africa: learning from the past and present to be better prepared for future outbreaks. Front Public Health. (2023) 11:1049986. doi: 10.3389/fpubh.2023.1049986 37228735 PMC10203177

[3] Africa Centres for Disease Control and Prevention. Building testing capacity for epidemic-prone diseases (2023). Available at: https://africacdc.org/download/building-testing-capacity-for-epidemic-prone-diseases (Accessed February 5-12, 2024).

[4] Africa Centres for Disease Control and Prevention. Disease outbreaks: information on disease outbreaks from Africa CDC (2023). Available at: https://africacdc.org/disease-outbreak/ (Accessed February 5-12, 2024).

[5] Hayman DTS Barraclough RK Muglia LJ McGovern V Afolabi MO N'Jai AU. Addressing the challenges of implementing evidence-based prioritisation in global health. BMJ Glob Health. (2023) 8:e012450. doi: 10.1136/bmjgh-2023-012450, PMID: 37290897 PMC10255200

[6] Adebisi YA Rabe A Lucero-Prisno DE III. COVID-19 surveillance systems in African countries. Health Promot Perspect. (2021) 11:382–92. doi: 10.34172/hpp.2021.49, PMID: 35079582 PMC8767077

[7] Aborode AT Hasan MM Jain S Okereke M Adedeji OJ Karra-Aly A. Impact of poor disease surveillance system on COVID-19 response in Africa: time to rethink and rebuilt. Clin Epidemiol Glob Health. (2021) 12:100841. doi: 10.1016/j.cegh.2021.100841, PMID: 34368503 PMC8330137

[8] Africa Centres for Disease Control and Prevention. Risk ranking and prioritization of epidemic-prone diseases (2023). Available at: https://africacdc.org/download/risk-ranking-and-prioritization-of-epidemic-prone-diseases/ (Accessed February 5-12, 2024).

[9] Nachega JB Nsanzimana S Rawat A Wilson LA Rosenthal PJ Siedner MJ. Advancing detection and response capacities for emerging and re-emerging pathogens in Africa. Lancet Infect Dis. (2023) 23:e185–9. doi: 10.1016/S1473-3099(22)00723-X, PMID: 36563700 PMC10023168

[10] Ewert B Wallenburg I Winblad U Bal R. Any lessons to learn? Pathways and impasses towards health system resilience in post-pandemic times. Health Econ Policy Law. (2023) 18:66–81. doi: 10.1017/S1744133122000238, PMID: 36121039

[11] Rajapaksha R Khatri RB Abeysena C Wijesinghe MSD Endalamaw A Thomas TK. Success and challenges of health systems resilience-enhancing strategies for managing public health emergencies of international concerns (PHEIC): a systematic review protocol. BMJ Open. (2022) 12:e067829. doi: 10.1136/bmjopen-2022-067829, PMID: 36410836 PMC9680175

[12] Jamison DT Gelband H Horton S Jha P Laxminarayan R Mock CN eds. Disease control priorities: Improving health and reducing poverty. Washington (DC): The World Bank (2017). 30212058

[13] Nyaruaba R Okoye CO Akan OD Mwaliko C Ebido CC Ayoola A. Socio-economic impacts of emerging infectious diseases in Africa. Infect Dis (Lond). (2022) 54:315–24. doi: 10.1080/23744235.2021.2022195, PMID: 35007473

[14] Craven M Sabow A Van der Veken L Wilson M. Not the last pandemic: investing now to reimagine public-health systems (2021). Available at: https://www.mckinsey.com/industries/public-sector/our-insights/not-the-last-pandemic-investing-now-to-reimagine-public-health-systems

[15] Balogun OO Akande OW Hamer DH. Lassa fever: an evolving emergency in West Africa. Am J Trop Med Hyg. (2020) 104:466–73. doi: 10.4269/ajtmh.20-0487, PMID: 33236712 PMC7866331

[16] Brehm B. The case for investment: insights from five African manufacturers on local manufacturing of diagnostic tools2023. Available at: https://www.path.org/our-impact/articles/investment-insights-local-manufacturing-diagnostic/ (Accessed February 5-12, 2024).

[17] The Global Fund. Results report 2023. Geneva, Switzerland: The Global Fund (2023).

[18] Ramos PIP Marcilio I Bento AI Penna GO de Oliveira JF Khouri R. Combining digital and molecular approaches using health and alternate data sources in a next-generation surveillance system for anticipating outbreaks of pandemic potential. JMIR Public Health Surveill. (2024) 10:e47673. doi: 10.2196/47673, PMID: 38194263 PMC10806444

[19] Calderaro A Piccolo G Chezzi C. The laboratory diagnosis of malaria: a focus on the diagnostic assays in non-endemic areas. Int J Mol Sci. (2024) 25:695. doi: 10.3390/ijms25020695, PMID: 38255768 PMC10815132

[20] African Society for Microbiology. Bringing diagnostic testing closer to communities. Lab Culture (2022). Available at: https://aslm.org/wp-content/uploads/2022/06/ASLM_Lab_Culture_Issue_27.pdf

[21] Kobia F Gitaka J. COVID-19: are Africa's diagnostic challenges blunting response effectiveness? AAS Open Res. (2020) 3:4. doi: 10.12688/aasopenres.13061.1, PMID: 32399515 PMC7205356

[22] Heidt B Siqueira WF Eersels K Diliën H van Grinsven B Fujiwara RT. Point of care diagnostics in resource-limited settings: a review of the present and future of PoC in its most needed environment. Biosensors (Basel). (2020) 10:133. doi: 10.3390/bios10100133, PMID: 32987809 PMC7598644

[23] Mfuh KO Abanda NN Titanji BK. Strengthening diagnostic capacity in Africa as a key pillar of public health and pandemic preparedness. PLOS Glob Public Health. (2023) 3:e0001998. doi: 10.1371/journal.pgph.0001998, PMID: 37310963 PMC10263319

[24] Chanda-Kapata P Ntoumi F Kapata N Lungu P Mucheleng'anga LA Chakaya J. Tuberculosis, HIV/AIDS and malaria health services in sub-Saharan Africa - a situation analysis of the disruptions and impact of the COVID-19 pandemic. Int J Infect Dis. (2022) 124:S41–s6. doi: 10.1016/j.ijid.2022.03.033 35341998 PMC8949686

